# Supplementary material for: Asthma and Post-Asthmatic Fibrosis: A Search for New Promising Molecular Markers of Transition from Acute Inflammation to Pulmonary Fibrosis
Source: Biomedicines. 2022 Apr 28;10(5):1017. doi: 10.3390/biomedicines10051017 (PMC9138542; doi:10.3390/biomedicines10051017)
Supplement: Supplementary file 1 [file biomedicines-10-01017-s001.zip › biomedicines-1683746-supplementary.pdf]

Materials and Methods

PPI networks reconstruction

The protein-protein interactions (PPI) were predicted based on data deposited in the Search Tool for the Retrieval of Interacting Genes/Genomes (STRING) database, with confidence score > 0.7. The reconstructed protein-protein pairs included functional relationships of proteins from five sources: published high-throughput experiments, genomic context prediction, co-expression, automated text mining, and PPI deposited in other databases. Reconstructed PPI networks were visualized as undirected networks using Cytoscape. To identify hub proteins most interconnected with their neighbors in the PPI network node degree scores were calculated, using Network Analyzer plugin [1], and degree centrality values of the nodes were visualized as heatmaps, using Morpheus tool (https://software.broadinstitute.org/morpheus/).

Data mining analysis

To analyze the co-occurrence of genes of interest and keywords associated with lung pathology in scientific texts deposited in MEDLINE database, a data-mining analysis of scientific literature was performed using GenClip3 web service [2]. The list of identified DEGs common for all analyzed asthma-associated and fibrosis-associated datasets was uploaded into GenClip3 and a search of co-occurrence of identified DEGs with the following keywords was performed: asthma, pulmonary fibrosis, lung fibrosis, hepatic fibrosis, liver fibrosis, renal fibrosis, kidney fibrosis.

ELISA

BAL fluids were analyzed for pro-inflammatory cytokines TNF-α and IL-6 by ELISA (Thermo Scientific, Rockford, IL, USA) according to the manufacturer's protocol. Briefly, 50 μL of BAL fluid and 50 μL of diluent were placed to wells with immobilised monoclonal antibodies against TNF-α or IL-6, filled with 50 μL of biotinylated anti-TNF-α or anti-IL-6 antibody solutions and incubated for 2 h at room temperature and 400 rpm in thermostatic shaker ST-3M (ELMI Ltd, Riga, Latvia). Next, the wells were sequentially filled with 100 μL of horseradish peroxidase labelled streptavidin (HRP-streptavidin) solution, 100 μL of 3,3′,5,5′-tetramethylbenzidine (TMB) solution and incubated for 1 h at room temperature and 400 rpm (for HPR-streptavidin) and 30 min at room temperature without direct light (for TMB). Before each addition, wells were washed five times with wash buffer. Stop solution was added to each well and the absorbance was measured at 450 nm using a Multiscan RC plate reader (Thermo Labsystems, Vantaa, Finland). The number of samples for ELISA assay was five for each experimental group.

References

1. Assenov, Y.; Ramírez, F.; Schelhorn S.E.S.E.; Lengauer, T., Albrecht, M. Computing topological parameters of biological networks. *Bioinformatics* **2008**, *24*, 282–4.

2. Wang, J.-H.; Zhao, L.-F.; Wang, H.-F.; Wen, Y.-T.; Jiang, K.-K.; Mao, X.-M.; Zhou, Z.-Y.; Yao, K.-T.; Geng, Q.-S.; Guo, D.; et al. GenCLiP 3: Mining human genes' functions and regulatory networks from PubMed based on co-occurrences and natural language processing. *Bioinformatics* **2019**, *36*, 1973–1975. https://doi.org/10.1093/bioinformatics/btz807.

Table S1. Characteristics of microarray datasets of murine and human lung pathologies.

GSE	Disease	Species	Source material	Platform
GSE27066	OVA-induced asthma	Mouse	Lung tissue	Affymetrix Mouse Genome 430 2.0 Array
GSE41665	OVA-induced asthma	Mouse	Lung tissue	Affymetrix Mouse Genome 430 2.0 Array
GSE116504	OVA-induced asthma	Mouse	Lung tissue	Agilent-074809 SurePrint G3 Mouse GE v2 8x60K Microarray
GSE122197	OVA-induced asthma	Mouse	Lung tissue	Agilent-074809 SurePrint G3 Mouse GE v2 8x60K Microarray
GSE50176	Carbon nanotubes induced asthma	Mouse	Lung tissue	Agilent-028005 SurePrint G3 Mouse GE 8x60K Microarray
GSE8553	Bleomycin-induced pulmonary fibrosis	Mouse	Lung tissue	Agilent-011978 Mouse Microarray G4121A
GSE25640	Bleomycin-induced pulmonary fibrosis	Mouse	Lung tissue	Affymetrix Mouse Genome 430 2.0 Array
GSE37635	Bleomycin-induced pulmonary fibrosis	Mouse	Lung tissue	Illumina MouseRef-8 v2.0 expression beadchip
GSE53845	Idiopathic pulmonary fibrosis	Human	Lung tissue	Agilent-014850 Whole Human Genome Microarray 4x44K G4112F
GSE24206	Idiopathic pulmonary fibrosis	Human	Lung tissue	Affymetrix Human Genome U133 Plus 2.0 Array
GSE72073	Idiopathic pulmonary fibrosis	Human	Lung tissue	Affymetrix Human Transcriptome Array 2.0
GSE33566	Idiopathic pulmonary fibrosis	Human	Peripheral blood	Agilent-014850 Whole Human Genome Microarray 4x44K G4112F
GSE103174	Chronic obstructive pulmonary disease	Human	Lung tissue	Affymetrix Human Genome U219 Array
GSE76925	Chronic obstructive pulmonary disease	Human	Lung tissue	Illumina HumanHT-12 V4.0 expression beadchip

GSE47460	Chronic obstructive pulmonary disease	Human	Lung tissue	Agilent-028004 SurePrint G3 Human GE 8x60K Microarray
GSE8581	Chronic obstructive pulmonary disease	Human	Lung tissue	Affymetrix Human Genome U133 Plus 2.0 Array
GSE29133	Chronic obstructive pulmonary disease	Human	Alveolar epithelial type II cells from lung tissue	HG-U133_Plus_2] Affymetrix Human Genome U133 Plus 2.0 Array
GSE100153	Chronic obstructive pulmonary disease	Human	Peripheral blood	Illumina HumanWG-6 v3.0 expression beadchip
GSE55962	Chronic obstructive pulmonary disease	Human	Total leukocytes from peripheral blood	[HG-U219] Affymetrix Human Genome U219 Array
GSE148004	Chronic obstructive pulmonary disease	Human	Sputum	Agilent-014850 Whole Human Genome Microarray 4x44K G4112F
GSE130928	Chronic obstructive pulmonary disease	Human	Alveolar macrophages from BAL	Affymetrix Human Genome U133 Plus 2.0 Array
GSE56341	Chronic obstructive pulmonary disease	Human	Epithelial cells of small airways from bronchial brushing	Affymetrix Human Gene 1.0 ST Array
GSE16972	Chronic obstructive pulmonary disease	Human	Alveolar macrophages from BAL	[HG-U133A] Affymetrix Human Genome U133A Array
GSE13896	Chronic obstructive pulmonary disease	Human	Alveolar macrophages from BAL	[HG-U133_Plus_2] Affymetrix Human Genome U133 Plus 2.0 Array
GSE1122	Chronic emphysema	Human	Lung tissue	Affymetrix Human Full Length HuGeneFL Array
GSE26296	Chronic emphysema	Human	Lung myeloid cells from lung tissue	Illumina HumanWG-6 v3.0 expression beadchip
GSE38267	Cystic fibrosis	Human	Peripheral blood	Agilent-028004 SurePrint G3 Human GE 8x60K Microarray
GSE40445	Cystic fibrosis	Human	Nasal epithelial cells	Custom Affymetrix HsAirway520108F array

Table S2. The primers used in the study.

Gene	Type	Sequence
Fn1	Forward	5′-CCAATTACGAACAAGACCAGAAG-3′
	Probe	((5,6)-FAM-5'-ACAGAGCACCATTGGAATTTCCGC-3'-BHQ1
	Reverse	5′-ACCCTCAGAAGTACAGTCGG-3′
Igf1	Forward	5′-TGGATGCTCTTCAGTTCGTG-3′
	Probe	5′-((5,6)-FAM)-TTGAAGTAAAAGCCCCTCGGTCCAC–BHQ1-3′
	Reverse	5′-AGTACATCTCCAGTCTCCTCAG-3′
Ccl2	Forward	5′-TCCACTACCTTTTCCACAACC-3′
	Probe	5′-((5,6)-FAM)-AAGGCATCACAGTCCGAGTCACAC–BHQ1-3′
	Reverse	5′-GGATCCACACCTTGCATTTAAG-3′
C3	Forward	5′-GTTTATTCCTTCATTTCGCCTGG-3′
	Probe	5′-((5,6)-FAM)-ACACCCTGATTGGAGCTAGTGGC–BHQ1-3′
	Reverse	5′-GATGGTTATCTCTTGGGTCACC-3′
Timp1	Forward	5′-CTCAAAGACCTATAGTGCTGGC-3′
	Probe	5′-((5,6)-FAM)-ACTCACTGTTTGTGGACGGATCAGG–BHQ1-3′
	Reverse	5′-CAAAGTGACGGCTCTGGTAG-3′
Cxcl12	Forward	5′-ACTCCAAACTGTGCCCTTC-3′
	Probe	5′-((5,6)-FAM)-ACAGACAAGTGTGCATTGACCCGA–BHQ1-3′
	Reverse	5′-GTCTACTGGAAAGTCCTTTGGG-3′
Ccl4	Forward	5′-AAACCTAACCCCGAGCAAC-3′
	Probe	5′- ((5,6)-FAM)- TTTCTCTTACACCTCCCGGCAGC-3′-BHQ1
	Reverse	5′-CTCCAAGTCACTCATGTACTCAG-3′
Ccr2	Forward	5′-GCTCTACATTCACTCCTTCCAC-3′
	Probe	5′-((5,6)-FAM)-CCCAACCGAGACCTCTTGCTCC–BHQ1-3′
	Reverse	5′-ACCACTGTCTTTGAGGCTTG-3′
Spp1	Forward	5′-CTACGACCATGAGATTGGCAG-3′
	Probe	5′-((5,6)-FAM)-AATCAGTCACTTTCACCGGGAGGG–BHQ1-3′
	Reverse	5′-TCTTCAGAGGACACAGCATTC-3′
Muc5b	Forward	5′-TGCCTATCAAAGTGTGGGAC-3′
	Probe	5′-((5,6)-FAM)-CTCGTAGTGGAAGTGGCAAGGCT-3′-BHQ1
	Reverse	5′-GAGCACGGAGGTACAGTTATC-3′
Ccr3	Forward	5′-AAAGGACTTAGCAAAATTCACCAG-3′
	Probe	5′-((5,6)-FAM)-CACACCCTATGAATATGAGTGGGCACC–BHQ1-3′
	Reverse	5′-AGTACAGTGGAGGCAGGAG-3′
C3ar1	Forward	5′-TTGGTCTCACTTGTCTATTGGG-3′

	Probe	5′-((5,6)-FAM)-ACCAGCCCATTGCCTAGCAGT–BHQ1-3′
	Reverse	5′-TCTTCATCTTTACGCCAGCTAC-3′
	Forward	5′-GAGTGACAGCAAGATGGAGG-3′
Muc5ac	Probe	5'-((5,6)-FAM-CCCACAAAAGCACCAGGCCAAT-3'-BHQ1
	Reverse	5′-TCATCAAAGTTCCCACACAGG-3′
	Forward	5′-AGTTGGTGCTAAGGGTGAAG-3′
Col1a1	Probe	5′- ((5,6)-FAM)-CTCTGAAGGTCCCCAGGGTGTG-3′-BHQ1
	Reverse	5′-TTTAGCGCCAGGTTGTCC-3′
	Forward	5′-CAGGTTTGACAGGTGAAGTTG-3′
Col4a1	Probe	5′-((5,6)-FAM)-AAAGGTCAGAAAGGAGAGAGCTGCC-3′-BHQ1
	Reverse	5′-CTTTAGCCCCAGGTTGTCC-3′
	Forward	5′-ACGGACAGAAGGGTGAAAAG-3′
Col4a2	Probe	5′-((5,6)-FAM)-ACATAGGACAGCCAGGACCCAAC-3′-BHQ1
	Reverse	5′-ACAAGTGTGATGTCAGATGGG-3′
	Forward	5′-TGGAATCGGAGATGCTTGTG-3′
Thbs2	Probe	5′-((5,6)-FAM)-CCTTCTCATCGCTCACACCGTCATT-3′-BHQ1
	Reverse	5′-GTCTCCAACCTCATCCTTGTC-3′
	Forward	5'-CCCCAAAATGGTTAAGGTTGC-3'
HPRT	Probe	5'- ((5,6)-ROX)-CTTGCTGGTGAAAAGGACCT–BHQ2-3'
	Reverse	5'-AACAAAGTCTGGCCTGTATCC-3'

Table S3. Nodes related to airway remodeling and lung fibrosis in functional analysis of DEGs, specific for asthma and pulmonary fibrosis.

Shared name	Unique ID	Term p-value
ERK1 and ERK2 cascade	GO:0070371	2.753471183989485E-10
Focal Adhesion	WP:85	2.198444526229187E-3
Lung fibrosis	WP:3632	6.065721439363499E-10
animal organ regeneration	GO:0031100	8.813872698604554E-6
cell chemotaxis	GO:0060326	7.489013538527099E-18
cell migration	GO:0016477	9.228201348004883E-15
cellular extravasation	GO:0045123	5.352589466078558E-4
chemotaxis	GO:0006935	3.7915353342522355E-18
endopeptidase inhibitor activity	GO:0004866	8.357082637758637E-4
endothelial cell proliferation	GO:0001935	1.7878630354746127E-5
epithelial cell proliferation	GO:0050673	5.30091784902138E-7
granulocyte chemotaxis	GO:0071621	1.906415996362048E-16
growth factor activity	GO:0008083	1.0672978430812795E-3
leukocyte migration	GO:0050900	2.9299515582459377E-15
macrophage migration	GO:1905517	5.93565722992562E-7
monocyte chemotaxis	GO:0002548	7.418884603838374E-13
mononuclear cell migration	GO:0071674	1.2457579607338416E-10
muscle cell proliferation	GO:0033002	2.126976343124111E-4
myeloid leukocyte migration	GO:0097529	1.01313520210322E-16
negative regulation of angiogenesis	GO:0016525	2.33919988975246E-3
negative regulation of cell adhesion	GO:0007162	1.3661017402546213E-4
negative regulation of cell migration	GO:0030336	5.960759200941078E-4
negative regulation of proteolysis	GO:0045861	3.1953199881326826E-5
peptidase regulator activity	GO:0061134	3.993794906904515E-4
positive regulation of ERK1 and ERK2 cascade	GO:0070374	2.3148275865508656E-8
positive regulation of angiogenesis	GO:0045766	2.3310217145445597E-4
positive regulation of cell adhesion	GO:0045785	2.744507824944852E-6
positive regulation of cell migration	GO:0030335	5.255469579708225E-10
positive regulation of cell population proliferation	GO:0008284	2.3293566808415894E-10
positive regulation of cell-cell adhesion	GO:0022409	4.172205360296355E-4
positive regulation of epithelial cell proliferation	GO:0050679	8.953565113467056E-4
positive regulation of leukocyte cell-cell adhesion	GO:1903039	5.454172638948849E-3
positive regulation of monocyte chemotaxis	GO:0090026	5.39065779500031E-6

positive regulation of mononuclear cell migration	GO:0071677	6.3276150059171E-8
positive regulation of wound healing	GO:0090303	1.769781679467732E-4
regeneration	GO:0031099	2.8164374974844117E-5
regulation of ERK1 and ERK2 cascade	GO:0070372	1.3808360105176887E-9
regulation of angiogenesis	GO:0045765	1.9514724762315453E-5
regulation of cell adhesion	GO:0030155	3.3716547663408435E-8
regulation of cell migration	GO:0030334	2.678285617871147E-10
regulation of cell-cell adhesion	GO:0022407	1.2419002294846432E-5
regulation of cell-substrate adhesion	GO:0010810	1.524413641192575E-4
regulation of cellular extravasation	GO:0002691	3.552198582402178E-5
regulation of endothelial cell proliferation	GO:0001936	8.849921556855754E-6
regulation of epithelial cell proliferation	GO:0050678	4.157817249847388E-6
regulation of granulocyte chemotaxis	GO:0071622	1.8937483045755902E-4
regulation of leukocyte cell-cell adhesion	GO:1903037	1.0374167489837815E-3
regulation of leukocyte migration	GO:0002685	1.4811836292154383E-8
regulation of smooth muscle cell migration	GO:0014910	1.1634669774507897E-4
regulation of smooth muscle cell proliferation	GO:0048660	2.4033285120932452E-4
regulation of vascular endothelial growth factor production	GO:0010574	8.624504908392829E-7
response to wounding	GO:0009611	1.903281051943597E-9
smooth muscle cell migration	GO:0014909	1.6900802885999375E-4
tube development	GO:0035295	8.747067340548854E-10
vascular endothelial growth factor production	GO:0010573	1.4820898538981286E-6
